# Predictive modeling based on tumor spectral CT parameters and clinical features for postoperative complications in patients undergoing colon resection for cancer

**DOI:** 10.1186/s13244-023-01515-5

**Published:** 2023-09-23

**Authors:** Xiaoying Tan, Xiao Yang, Shudong Hu, Xingbiao Chen, Zongqiong Sun

**Affiliations:** 1 Department of Radiology, Binhu District, Affiliated Hospital of Jiangnan University, Hefeng Road 1000#, Wuxi City, 214062 Jiangsu Province China; 2 Department of Clinical Science, Philips Healthcare, Shanghai, 200233 China

**Keywords:** Colon cancer, Postoperative complications, Spectral CT, Combined model, Prediction

## Abstract

**Background:**

Colon cancer is a particularly prevalent malignancy that produces postoperative complications (POCs). However, limited imaging modality exists on the accurate diagnosis of POCs. The purpose of this study was therefore to construct a model combining tumor spectral CT parameters and clinical features to predict POCs before surgery in colon cancer.

**Methods:**

This retrospective study included 85 patients who had preoperative abdominal spectral CT scans and underwent radical colon cancer resection at our institution. The patients were divided into two groups based on the absence (no complication/grade I) or presence (grades II–V) of POCs according to the Clavien-Dindo grading system. The visceral fat areas (VFA) of patients were semi-automatically outlined and calculated on L3-level CT images using ImageJ software. Clinical features and tumor spectral CT parameters were statistically compared between the two groups. A combined model of spectral CT parameters and clinical features was established by stepwise regression to predict POCs in colon cancer. The diagnostic performance of the model was evaluated using the receiver operating characteristic (ROC) curve, including area under the curve (AUC), sensitivity, and specificity.

**Results:**

Twenty-seven patients with POCs and 58 patients without POCs were included in this study. MonoE_40keV-VP_ and VFA were independent predictors of POCs. The combined model based on predictors yielded an AUC of 0.84 (95% CI: 0.74–0.91), with a sensitivity of 77.8% and specificity of 87.9%.

**Conclusions:**

The model combining MonoE_40keV-VP_ and VFA can predict POCs before surgery in colon cancer and provide a basis for individualized management plans.

**Critical relevance statement:**

The model combining MonoE40keV-VP and visceral fat area can predict postoperative complications before surgery in colon cancer and provide a basis for individualized management plans.

**Key points:**

• Visceral fat area and MonoE40keV-VP were independent predictors of postoperative complications in colon cancer.

• The combined model yielded a high AUC, sensitivity, and specificity in predicting postoperative complications.

• The combined model was superior to the single visceral fat area or MonoE40keV-VP in predicting postoperative complications.

**Graphical Abstract:**

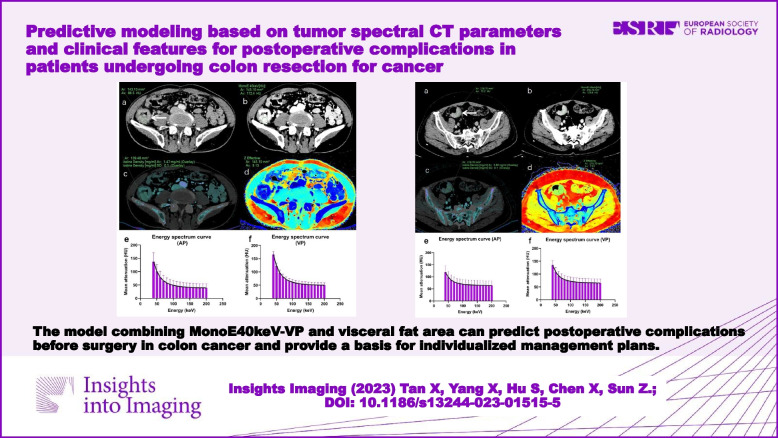

## Background

Colon cancer is the third most common cancer in the world and the second leading cause of cancer-related death [1]. Although chemotherapy and immunotherapy are now widely used as neo-adjuvant or adjuvant treatments in colon cancer patients, surgery is still the only curative treatment [2]. However, postoperative complications (POCs) can seriously affect the prognosis of patients and reduce their overall survival [3]. Therefore, it is important to effectively predict the occurrence of POCs in colon cancer.

To the best of our knowledge, all studies performed so far found that visceral obesity is an independent predictor of POCs in colon cancer, and obese patients are more likely to have complications, such as anastomotic fistula and wound infection [4–7]. Currently, CT is primarily used for measuring visceral fat, reflecting whole-body fat content through the measurement of fat at the L3-L5 level [8, 9]. However, the predictability of POCs using visceral fat and clinicopathological information alone is inefficient, with AUCs of less than 0.80 [10, 11]. Therefore, it is important to construct a new, more valid, and objective prediction method.

Colon cancer has different histopathological subtypes, with heterogeneities within and between tumors. Intra-tumor heterogeneity may contribute to different subtypes and various gene expressions within the tumor, which may affect the treatment outcome [12]. Contrast-enhanced CT of the abdomen by intravenous contrast injection is the main imaging tool for the preoperative assessment of colon cancer, evaluating the tumor stage and distant metastases, as well as the density and distribution of angiogenesis within the tumor. The enhanced image reflects the different degrees of enhancement between different histological types of tumors, thus demonstrating intra-tumoral heterogeneities [13]. CT has good spatial resolution and assesses the interior of the tumor without being limited by the spatial heterogeneity of the tumor, as demonstrated in a study of colorectal cancer using preoperative CT colonography [14].

CT technology has advanced rapidly, and the emerging spectral CT imaging technology can provide more quantitative information about the inside of tumors. Combining quantitative and qualitative information, spectral CT can provide virtual monoenergetic images (MonoE) to enhance image contrast, an iodine density map can quantify the iodine content inside the tumor that reflects the density of blood vessels, effective atomic number map can reflect on material information, which is mostly applied to stone composition, and the energy spectrum curve can reflect on the degree of attenuation of the material [15–18]. Spectral CT has been used in diagnosing lymph node metastasis and lymphovascular invasion [19, 20]. However, to the best of our knowledge, there have been no studies on the application of spectral CT in POCs of colon cancer.

Therefore, this study aimed to establish a predictive model combining clinical features and tumor spectral CT parameters for the effective prediction of POCs in colon cancer to develop individualized treatment plans.

## Materials and methods

### Patients

**Fig. 1:**
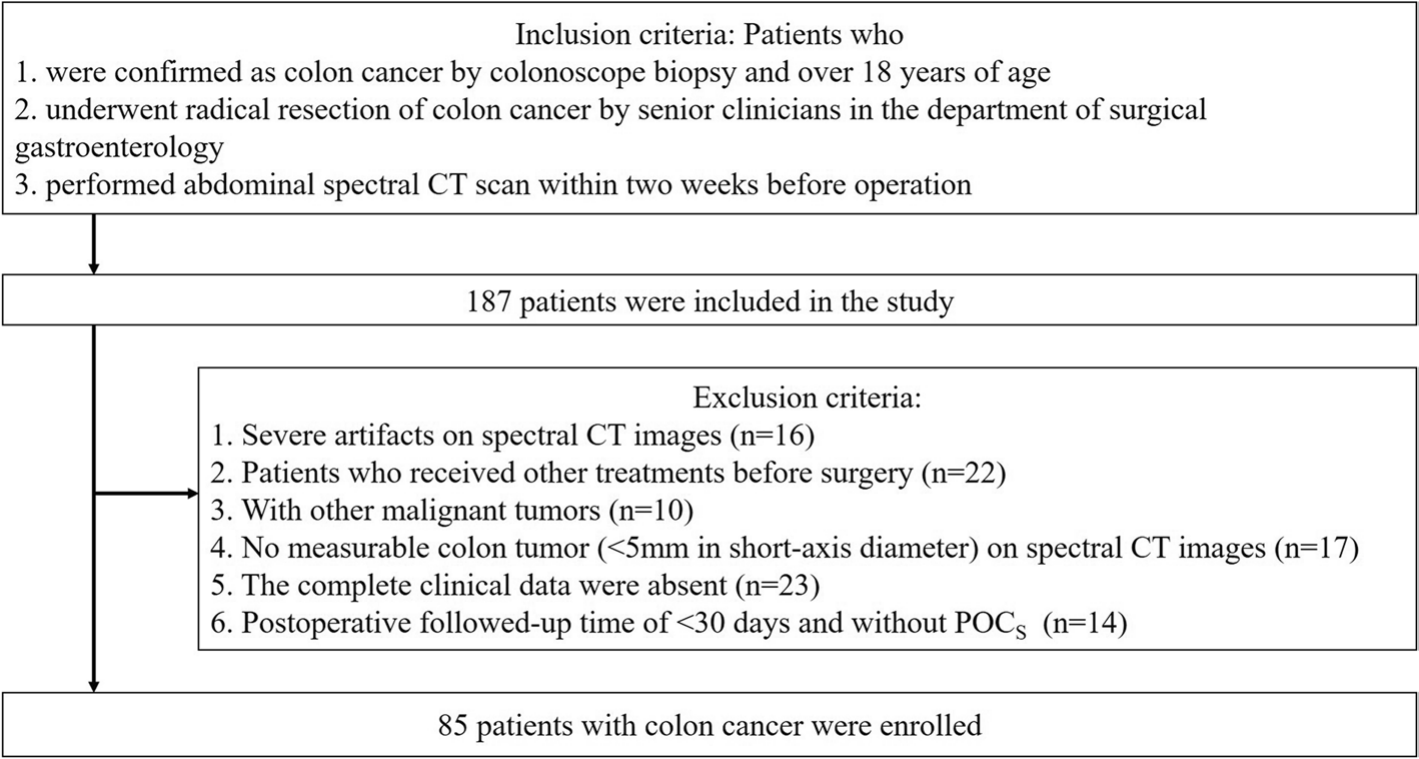
Flow diagram of patient selection

This retrospective study initially included 187 consecutive patients with pathologically confirmed colon cancer postoperatively from picture archiving and communication systems (PACS) in our hospital from August 2021 to March 2023. The inclusion criteria were as follows: (1) patients over 18 years of age who underwent radical colon cancer surgery, including the removal of the colonic segment harboring the tumor with proximal and distal 10 cm margins, complete mesocolic excision (CME) with central vascular ligation and D3 lymphadenectomy; (2) abdominal spectral CT imaging scans were performed within two weeks before surgery; (3) the surgical procedures were performed by senior clinicians and nurses with more than 10 years of experience in treating colon cancer in the department of surgical gastroenterology. The exclusion criteria were as follows: (1) CT images with poor quality and large lesion artifacts; (2) patients who had preoperative radiotherapy or neoadjuvant chemotherapy; (3) patients with a short diameter of the colon tumor of < 5 mm, which might have prevented the placement of ROI and impacted the measurement of intra-tumor spectral CT parameters; (4) patients with other malignant tumors in the body, which might have affected the patient’s physical condition; (5) patients with incomplete clinicopathological data; (6) postoperative follow-up time of < 30 days and without POCs. Finally, eighty-five patients with colon cancer were selected for further analysis, as shown in the patient selection flow chart (Fig. 1). POCs in colon cancer were dichotomized according to the Clavien-Dindo grading system [21], defining grades II–V as cancer with POCs and grades I and no complications as cancer without POCs [22]. The tumor-node-metastasis (TNM) staging of colon cancer was performed according to the criteria outlined in the 8th edition of the American Joint Committee on Cancer (AJCC)’s cancer staging manual [23]. This included stage I (T_1-2_N_0_M_0_), stage II (T_3-4_N_0_M_0_), stage III (T_any_N_1-2_M_0_), and stage IV (T_any_N_any_M_1_). Epidermal growth factor receptor (EGFR) expression levels of tumor were determined by immunohistochemical examination from the department of pathology. Clinical baseline characteristics of these two groups of patients were recorded from digital clinical files of PACS.

### Spectral CT image acquisition

All patients underwent abdominal spectral CT scanning (IQon Spectral CT, Philips Healthcare, Best, the Netherlands) with an unenhanced and dual-phase enhanced scan. The main parameters were tube voltage of 120 kVp, automatic tube current modulation, collimation of 64 × 0.625 mm, pitch value of 0.99, X-ray tube rotation speed of 0.75 s, and the reconstruction slice thickness of 1.0 mm. A contrast enhancement scan was performed with a fully automatic high-pressure syringe by injecting 80 mL of iodixanol (320 mgI/mL) through the elbow vein at a rate of 3.0 mL/s. The scan was triggered at the level of the coeliac trunk of the abdominal aorta in the arterial phase with a threshold of 150 HU. The scan was delayed by 70 s in the venous phase. Both conventional images and spectral-based images (SBI) data were reconstructed.

### Assessment of fat distribution on CT images

**Fig. 2:**
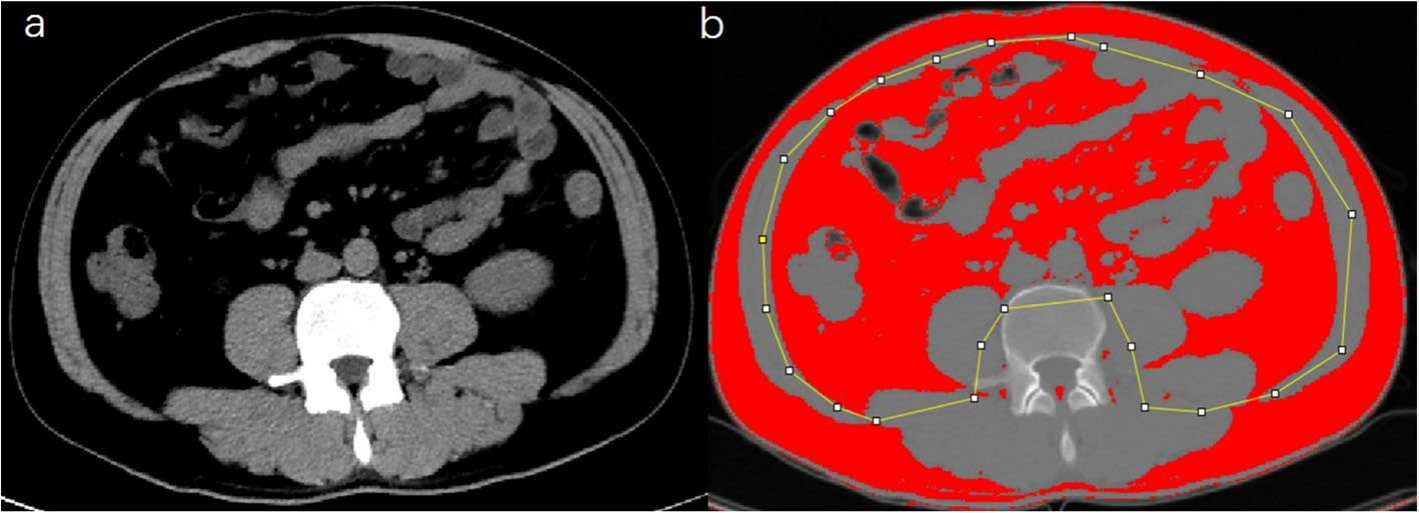
Measurement schematic diagram of VFA and SFA based on L3 level slice CT axial image. **a** Before outlining and delineating subcutaneous adipose tissue (SAT) and visceral adipose tissue (VAT). **b** After outlining and delineating SAT and VAT. VFA, visceral fat area; SFA, subcutaneous fat area

Visceral fat area (VFA) and subcutaneous fat area (SFA) were measured semi-automatically on L3 horizontal cross-sectional unenhanced CT images (plain scans were obtained before contrast-enhanced images) by a radiologist with 10 years of experience in the diagnosis of abdominal spectral CT imaging who was unknown about the patient’s outcome, using ImageJ software, as shown in the schematic diagram (Fig. 2). The CT threshold range for the adipose tissue was set as − 150 HU to − 50 HU for the visceral fat and − 190 to − 30 HU for the subcutaneous fat. The ratio of VFA/SFA determined the distribution of fat.

### Acquisition of tumor energy spectrum parameters

The spectral CT images, including virtual monoenergetic images (MonoE), iodine density (IoD), Z effective atomic number (Z-eff), and the energy spectrum curve, were generated from spectral base images (SBI) data package via post-processing workstation (IntelliSpace Portal 10, Philips Healthcare). Two radiologists with 8 and 10 years of experience in diagnostic imaging, respectively, opened thin-layer (1 mm layer thickness) spectral CT images on a Philips post-processing workstation ISP (Image and information management software version 10.1.5) in the arterial phase (AP) and venous phase (VP) and drew a circular ROI on the largest cross-sectional AP image of the colon tumor, avoiding areas of necrosis visible to the naked eye, and the ROI was copied onto the VP image with manual fine-tuning. Both physicians were unaware of the patient's postoperative results and recorded the values of the respective parameters of the colon tumor, such as conventional CT value (C) of the AP and VP, MonoE_40keV-AP_, MonoE_40keV-VP_, IoD_AP_, IoD_VP_, Z-eff_AP_, and Z-eff_VP_. The arterial enhancement fraction (AEF) values of colon cancer were further calculated using the formula of AEF = IoD_AP_/IoD_VP_. The slope of the energy spectrum curve (λ) was calculated as (CT40 kev-CT70 kev)/(70–40). The average of all colorectal tumor spectral CT parameters measured by two physicians was taken as the final recorded value.

### Statistical analysis

Statistical analyses were performed using SPSS 26.0 software (SPSS Inc., Chicago, IL, USA), MedCalc software version 15.11.4 (Marillac, Belgium), and R software (version 3.5.1; http://www.R-projetc.org). Interobserver agreements on the tumor spectral CT parameters were tested by the intraclass correlation coefficient (ICC), and with ICC > 0.75 indicating a good consistency. Quantitative data conforming to a normal distribution was expressed as mean ± standard deviation, an independent samples *t*-test was used to compare cancer with POCs and cancer without POCs, and those not normally distributed were expressed as median (upper quartile; lower quartile), and the Mann–Whitney *U* test was used for the comparison between the two groups. Qualitative information was expressed as frequency (percentage), and the comparison between the two groups was done using the appropriate chi-square test or Fisher’s exact test. The clinical features and spectral CT parameters that were statistically different were analyzed by stepwise regression to obtain independent predictors to POCs and build a predictive joint model. The diagnostic performance of the prediction model, including area under the curve (AUC), sensitivity, and specificity, was assessed using the receiver operating characteristic (ROC) curve. A *p* value of < 0.05 was considered statistically significant between the two groups.

## Results

### Patient clinical characteristics

In the recruited 85 colon cancer patients, there were 60 men and 25 women, with an age range of 30–85 years and a median age of 65 years. Twenty-seven patients (31.8%) were with POCs and 58 patients (68.2%) were without POCs on the basis of clinician’s diagnosis of postoperative medical history records from PACS database and the Clavien-Dindo grading system. POCs included infections (*n* = 17), such as lung infection (*n* = 3), abdominal infection (*n* = 10), and incisional infection (*n* = 4); anastomotic fistula (*n* = 2), pancreatic leakage (*n* = 3), multiple organ failure (*n* = 2), and blood transfusion and total parenteral nutrition (*n* = 3). When comparing the clinical baseline characteristics of the two groups, statistical differences were found in gender, EGFR expression, VFA, and VFA/SFA (all *p* < 0.05); the rest of the clinical characteristics were not statistically different between the two groups (all *p* > 0.05). The detailed results are shown in Table 1.

**Table 1:** Comparisons of clinical baseline characteristics between two groups of colon cancer patients (*n* = 85)

Clinical characteristics	Without POCs	With POCs	z/χ^2^ value	*p* value
	*n* = 58	*n* = 27		
Age (years)^c^	65 (56, 70)	68 (62, 74)	− 1.318^a^	0.188
Gender			4.061^b^	0.044^*^
Male	37 (63.8)	23 (85.2)		
Female	21 (36.2)	4 (14.8)		
Anemia			0.000^b^	1
Yes	10 (17.2)	4 (14.8)		
No	48 (82.8)	23 (85.2)		
Diabetes			0.002^b^	0.961
Yes	11 (19.0)	5 (18.5)		
No	47 (81.0)	22 (81.5)		
Hypertension			0.280^b^	0.597
Yes	25 (43.1)	10 (37.0)		
No	33 (56.9)	17 (63.0)		
TNM stage			4.927^b^	0.164
I	14 (24.1)	7 (25.9)		
II	21 (36.2)	4 (14.8)		
III	20 (34.5)	13 (48.2)		
IV	3 (5.2)	3 (11.1)		
Degree of cell differentiation			2.567^b^	0.109
Poor	12 (20.7)	10 (37.0)		
Moderate	46 (79.3)	17 (63.0)		
VFA (cm^2^)^c^	122.74 (83.09, 138.52)	158.20 (147.84, 168.76)	− 3.880^a^	0.000^*^
VFA/SFA^c^	0.80 (0.59, 1.09)	1.28 (0.92, 1.84)	− 3.747^a^	0.000^*^
Albumin (g/L)			0.706^b^	0.401
< 35	18 (31.0)	6 (22.2)		
> 35	40 (69.0)	21 (77.8)		
HER-2			0.292^b^	0.589
Positive	3 (5.2)	3 (11.1)		
Negative	55 (94.8)	24 (88.9)		
Ki-67			0.001^b^	0.974
Positive	52 (89.7)	25 (92.6)		
Negative	6 (10.3)	2 (7.4)		
EGFR			3.931^b^	0.047^*^
Positive	40 (68.9)	24 (88.9)		
Negative	18 (31.1)	3 (11.1)		

*POCs* postoperative complications, *TNM* tumor node metastasis, *VFA* visceral fat area, *SFA* subcutaneous fat area, *HER-2* human epidermal growth factor receptor 2, *EGFR* epidermal growth factor receptor

^*^Values denote *p* < 0.05 and the difference is statistically significant

^a^Value indicates that the statistic is* z*-value

^b^Values express that the statistic is *χ*^2^ value

^c^Values are expressed as median (upper quartile; lower quartile), other values are expressed as number (percentage)

### Tumor energy spectrum CT parameters

**Fig. 3:**
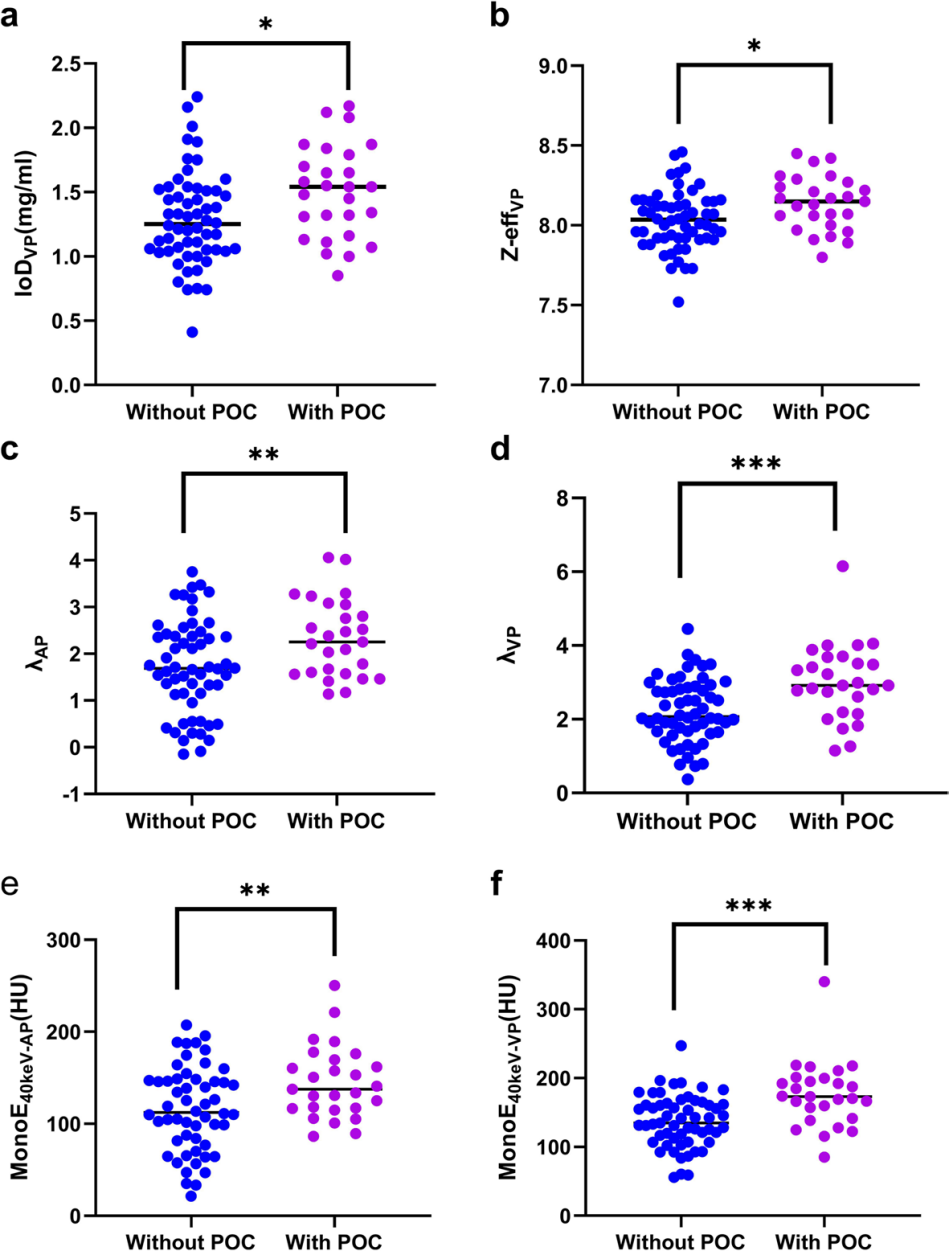
Scatter plot and comparison of tumor spectral CT parameters between colon cancer patients with POC_S_ and without POC_S_: IoD_VP_ (**a**), Z-eff_VP_ (**b**), λ_AP_ (**c**), λ_VP_ (**d**), MonoE_40kev-AP_ (**e**), MonoE_40keV-VP_ (**f**). *p < 0.05, **p < 0.01, and ***p < 0.001. POC, postoperative complications; IoD, iodine density; Z-eff, Z effective atomic number; MonoE, virtual monoenergetic images; AP, arterial phase; VP, venous phase; λ, spectral curve slope

**Fig. 4:**
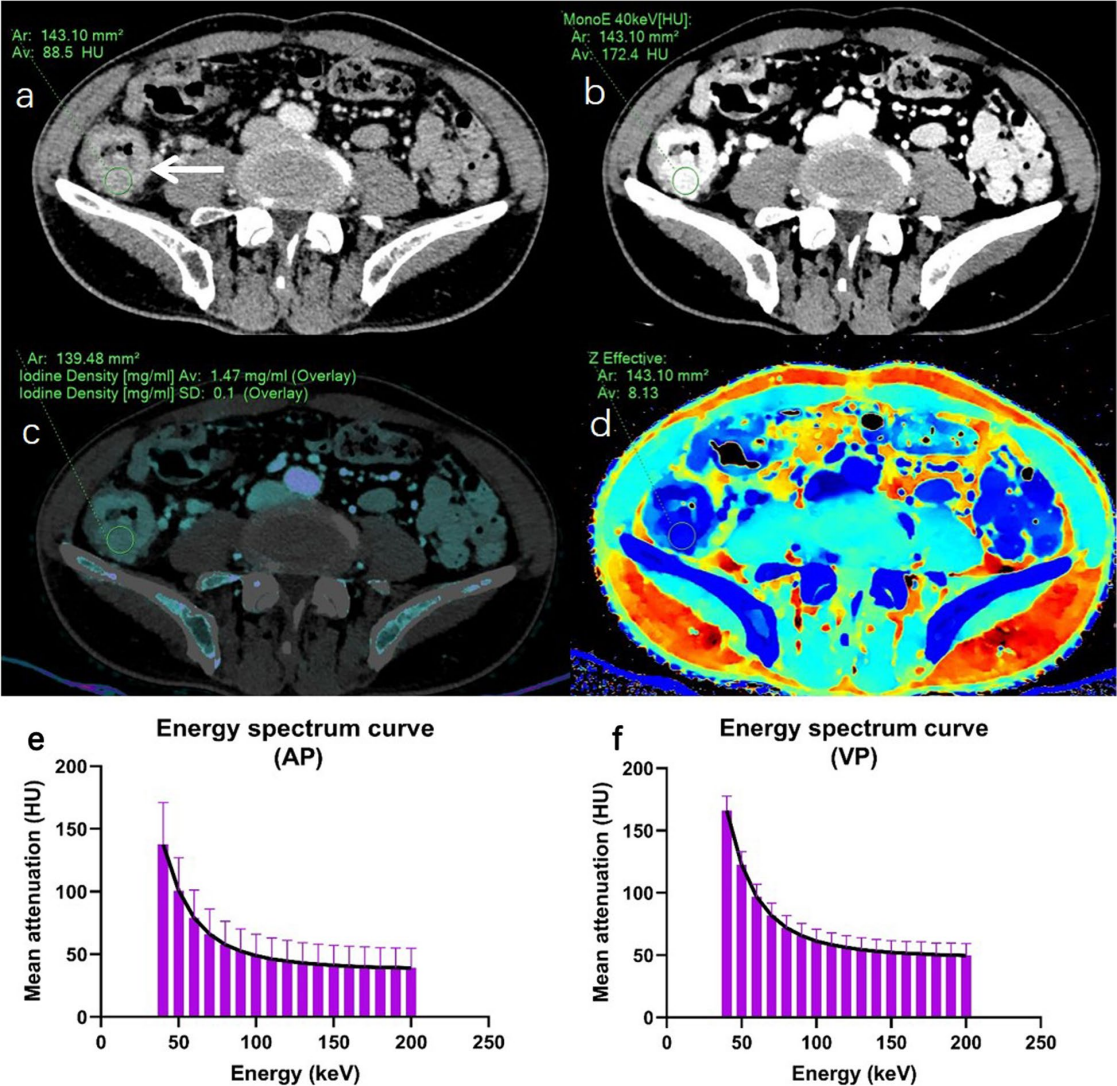
A 64-year-old colon cancer man with postoperative complications. Axial conventional CT image (**a**), MonoE_40keV-VP_ (**b**), iodine density (IoD) (**c**), and Z effective (Z-eff) atomic number images (**d**) in the venous phase (VP) show a mass (circular ROI) in the right colon, and their values in tumor ROI are respectively 88.5 HU, 172.4 HU, 1.47 mg/mL, and 8.13. The slope of spectral curve is 2.38 in the arterial phase (AP) (**e**). The slope of spectral curve is 2.81 in the VP (**f**)

**Fig. 5:**
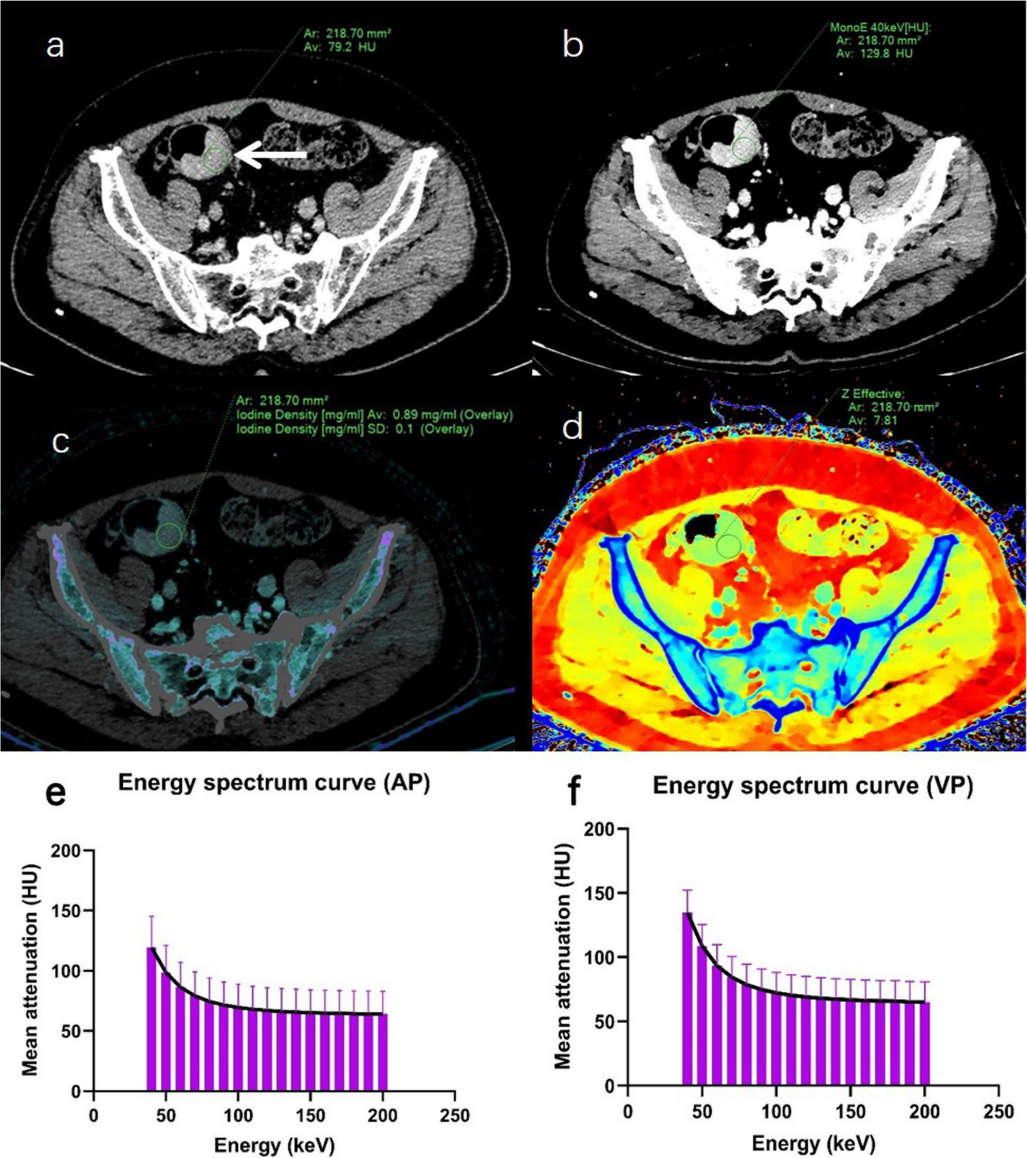
A 71-year-old colon cancer man without postoperative complications. Axial conventional CT image (**a**), MonoE_40keV-VP_ (**b**), iodine density (IoD) (**c**), and Z effective (Z-eff) atomic number images (**d**) in the venous phase (VP) show a mass (circular ROI) in the sigmoid colon, and their values in tumor ROI are respectively 79.2 HU, 129.8 HU, 0.89 mg/mL, and 7.81. The slope of spectral curve is 1.33 in the arterial phase (AP) (**e**). The slope of spectral curve is 1.68 in the VP (**f**)

The interobserver agreements for all tumor spectral CT parameters measured by two radiologists were good, with ICC values exceeding 0.85 (Table 2). When comparing the tumor spectral CT parameters between the two groups of colon cancer patients, MonoE_40keV-AP_, λ_AP_, C_VP_, MonoE_40keV-VP_, IoD_VP_, Z-EFF_VP_, and λ_VP_ were statistically significant (all *p* < 0.05) (Fig. 3). Other tumor spectral CT parameters were not statistically different between the two groups of colon cancer patients (all *p* > 0.05) (Table 2). Two representative spectral CT images of colon cancer patients, including energy spectrum curves, are shown in Figs. 4 and 5.

**Table 2:** Comparisons of tumor spectral CT parameters between two groups of colon cancer patients, and ICC between two radiologists’ measurements of parameters

Tumor spectral CT parameters	Without POCs	With POCs	*t*	*p*	ICC
	(*n* = 58)	(*n* = 27)			
C_AP_ (HU)	76.89 ± 15.44	78.31 ± 15.57	− 0.393	0.695	0.907
MonoE_40kev-AP_ (HU)	115.91 ± 44.79	144.95 ± 48.39.38	− 2.888	0.005^*^	0.890
IoD_AP_ (mg/mL)	1.10 ± 0.42	1.18 ± 0.46	− 0.777	0.440	0.912
Z-eff_AP_	7.93 ± 0.23	7.97 ± 0.26	− 0.723	0.472	0.901
λ_AP_	1.71 ± 1.00	2.33 ± 0.83	− 2.795	0.006^*^	0.889
C_VP_ (HU)	80.29 ± 11.52	85.56 ± 10.50	− 2.014	0.047^*^	0.893
MonoE_40kev-VP_ (HU)	138.06 ± 37.78	175.91 ± 47.57	− 3.953	0.000^*^	0.904
IoD_VP_ (mg/mL)	1.29 ± 0.36	1.50 ± 0.36	− 2.518	0.014^*^	0.897
Z-eff_VP_	8.03 ± 0.18	8.14 ± 0.17	− 2.574	0.012^*^	0.886
λ_VP_	2.23 ± 0.86	3.01 ± 1.03	− 3.678	0.000^*^	0.915
AEF	0.88 ± 0.31	0.77 ± 0.27	1.497	0.138	0.898

Values are expressed as mean ± standard deviation

*POCs* postoperative complications, *ICC* intraclass correlation coefficient, *C* conventional CT, *MonoE* virtual monoenergetic images, *IoD* iodine density, *AP* arterial phase, *VP* venous phase, *Z-eff* Z effective atomic number, *AEF* arterial enhancement fraction, *λ* spectral curve slope

^*^Values denote *p* < 0.05

### Evaluation of prediction model diagnosis efficacy of POCs

**Fig. 6:**
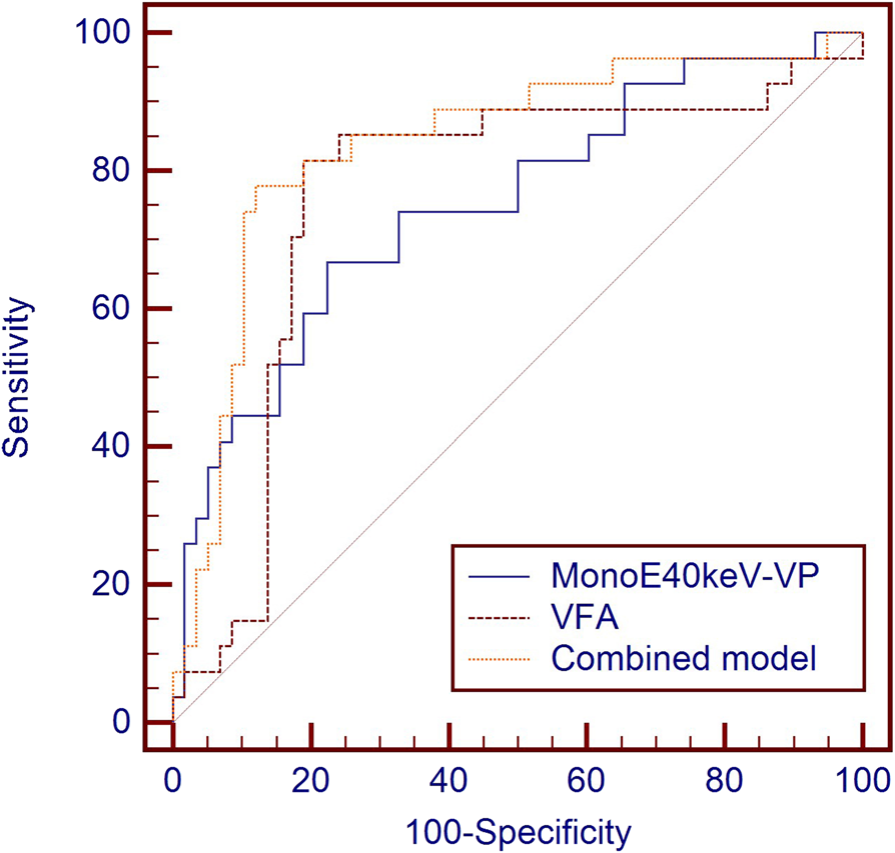
Performance of MonoE_40keV-VP_, VFA, and combined model in predicting postoperative complications in colon cancer by ROC analyses. ROC, receiver operating characteristic curve; MonoE, virtual monoenergetic image; VP, venous phase; VFA, visceral fat area

Stepwise regression analyses found that clinical characteristics VFA [odd ratio (OR): 1.021 (95%CI: 1.007–1.035); *p* = 0.002], and tumor spectral CT parameter MonoE_40keV-VP_ [OR: 1.026 (95%CI: 1.011–1.041); *p* = 0.001] were independent predictors of POCs in colon cancer, and a predictive joint model established from these two predictors yielded an AUC of 0.84 (95% CI: 0.74–0.91), with a sensitivity of 77.8%, and specificity of 87.9%. Similarly, VFA from clinical characteristics produced an AUC of 0.76 (95% CI: 0.66–0.85) with a sensitivity of 81.5% and specificity of 81%. The spectral CT parameters of MonoE_40keV-VP_ produced an AUC of 0.75 (95% CI: 0.64–0.84), with a sensitivity of 66.7% and specificity of 77.6% (Table 3 and Fig. 6).

**Table 3:** Diagnostic performance of MonoE_40keV-VP_, VFA, and combined model in predicting POCs in patients with colon cancer by ROC analyses

Prediction model	AUC (95% CI)	Accuracy	Sensitivity	Specificity
MonoE_40keV-VP_	0.75 (0.64–0.84)	74.1%	66.7%	77.6%
VFA	0.76 (0.66–0.85)	81.2%	81.5%	81.0%
Combined model	0.84 (0.74–0.91)	84.7%	77.8%	87.9%

*POCs* postoperative complications, *MonoE* virtual Monoenergetic images, *VFA* visceral fat area, *ROC* receiver operating characteristic curve, *AUC* area under the curve, *CI* confidence interval

## Discussion

POCs in colon cancer were associated with the spectral CT parameter MonoE_40keV-VP_ based on an AUC of 0.75. Further, the clinical characteristic VFA was associated with POCs in colon cancer. Subsequently, a novel combined prediction model was constructed by incorporating two independent predictor variables, such as MonoE_40keV-VP_ and VFA through stepwise regression and yielded an AUC of 0.84 with a sensitivity of 77.8% and specificity of 87.9%, showing good predictive performance.

Specific to clinical characteristics, the VFA was an independent predictor of POCs in colon cancer. Patients with large VFA were more likely to have POCs, the greater the VFA/SFA value, the greater the distribution of visceral fat and the greater the risk of POCs for patients, which was consistent with previous studies [4–7]. However, both VFA and VFA/SFA mean the same thing (visceral fat). When we built a predictive joint model by stepwise regression method, because of the collinearity problem between them, VFA was included first, so VFA/SFA was automatically excluded from the model and was not an independent predictor of POCs. Previous studies showed that visceral obesity increased the incidence of rectal, esophageal, pancreatic, and hepatocellular cancers and that increased visceral adiposity induced chronic systemic inflammation and altered metabolic environments that promoted cancer development [24]. It also increased the incidence of POCs [25, 26]. Because chronic inflammatory factors were lower in obese tissue than those in acute immune activation, the ability to clear the tissue from infectious agents was reduced, and adipose tissue released pro-inflammatory factors, but this ability was reduced due to chronic overnutrition [27, 28]. Additionally, a previous study [29] reported that the occurrence of POCs was gender-related, with male patients being more likely to experience POCs. This finding was consistent with the results of the current study, which also observed a higher incidence of POCs in male patients. The gender difference in POCs was attributed to higher level of visceral fat, as they are more prone to abdominal fat accumulation compared to women [30]. Furthermore, a study demonstrated that patients with high visceral fat were at an increased risk of anastomotic leakage after colon cancer resection [26]. However, the present study could not confirm this association due to the small sample size, with only two cases of anastomotic fistula observed.

In recent years, predictive models for POCs in colon cancer were developed but the models were mainly based on the clinicopathological characteristics, including the VFA of the patients [7, 31]. Kuritzkes et al. [31] analyzed 264 patients who underwent radical resection for colorectal cancer using logistic regression and found that VFA was an independent risk factor for POCs (Clavien-Dindo grades ≥ III), with an AUC of 0.660; Heus et al. [7] analyzed 406 patients with colorectal cancer and found that preoperative body composition parameters were highly correlated with the POCs, with patients with high VFA being more likely to have poor postoperative outcomes. In addition, gender and EGFR expression were found to be associated with POCs in our study; however, they were not independent predictor factors through the stepwise regression analysis. The prediction model based on VFA was developed with an AUC of 0.76, but the results were still not ideal.

Regarding tumor spectral CT parameters, spectral CT imaging reflected the blood supply to the tumor by quantifying the iodine content of the local tissue, thereby revealing intra-tumor heterogeneity and predicting patient prognosis [16]. In addition, the Z effective atomic number map was derived from the attenuation of X-rays, highlighting the difference in Z-eff values between the lesion and the surrounding tissue, indirectly reflecting tissue perfusion and revealing tumor heterogeneity [32]. Previous studies found that IoD in tumors was associated with lymph node metastasis in colon cancer as well as tumor staging [16, 17]. The tumor’s Z-eff, λ, and MonoE values identified mutant versus wild-type KRAS genes in colon cancer to stage and histologically grade tumors [18, 33]. However, there are no reports on the use of spectral CT parameters for the prediction of POCs in colon cancer. In this study, several spectral CT parameters were associated with POCs, and patients who had POCs in colon cancer had higher values of spectral CT parameters, including MonoE_40keV-AP_, λ_AP_, MonoE_40keV-VP_, ID_VP_, Z-eff_VP_, and λ_VP_. The higher the value of the spectral CT parameter of the tumor indicated the richer the blood supply in the tumor and the more pronounced enhancement. The richer blood supply was associated with faster tumor growth, and the more aggressive tumors were more likely to invade the surrounding tissues, thus affecting the patient’s survival and prognosis [34]. Furthermore, there are more tumor spectral CT parameters with significant statistical differences (*p* < 0.05) in VP than AP between patients with POCs and without POCs, which may be related to the delayed enhancement of colon cancer, and the features of the VP were more clearly related to the intensification of microvessels and better reflected the characteristics of the tumor, which was consistent with a previous study [35]. Spectral CT can provide more useful and valuable information compared to a single conventional CT parameter, which can provide richer information regarding the tumor. In this study, the spectral CT parameter MonoE_40keV-VP_ was the independent predictor and predicted POCs in colon cancer, yielding an AUC of 0.75, but the results were still not good.

Further, we found the patient’s clinical characteristic VFA, and tumor spectral CT parameter MonoE_40keV-VP_ as independent predictors of POCs in colon cancer by stepwise regression analysis, and a combined prediction model was established, which showed a better predictive effect (AUC = 0.84) with a sensitivity of 77.8% and specificity of 87.9%. Cao et al. [16] combined clinical features with energy spectrum parameters to construct a model that effectively predicted lymph node metastasis in colorectal cancer (AUC = 0.876). In this study, we constructed the first combined model based on clinical features and tumor spectral CT parameters to predict POCs in colon cancer, yielding good predictive power. The model can help in developing individualized treatment plans for patients undergoing radical colon cancer surgery, potentially leading to a reduction in the length of hospital stay.

This study still had some limitations. First, the small sample size of patients undergoing radical colon cancer in this study may have resulted in some selection bias. A larger cohort is requested. Second, this study only outlined the circular region of interest at the largest tumor level, which may have resulted in the loss of some biological information. A whole tumor mean calculation was yet to be performed to analyze whether there was a statistical difference from a single level. Third, this study was a single-center retrospective study and the prediction model we developed lacked external validation. A further multicenter prospective study is pending, aiming to optimize the generalizability and performance of the prediction model. Fourth, in this study, POCs were diagnosed on the basis of clinician’s diagnosis from medical records alone. Diagnoses such as multiple organ failure, blood transfusion and total parenteral nutrition may well be taken from medical records. However, the situation is different when it comes to infections where most cases are diagnosed not only on the basis of clinical signs, lab values, etc., but also based on imaging. This especially applies to complications such as abdominal infection, anastomotic fistula, and pancreatic leakage which were recorded in the medical records of some patients. So the lack of CT confirmation and/or re-evaluation of images in our retrospective study may have resulted in some bias.

## Conclusion

In conclusion, we constructed a combined model based on the patient’s VFA and the tumor spectral CT parameter MonoE_40keV-VP_, which well-predicted POCs in colon cancer underwent surgery and provided a basis for personalized treatment for colon cancer patients.

## Data Availability

The datasets generated during and/or analyzed during the current study are available from the corresponding author at reasonable request.

## Abbreviations

AEF: Arterial enhancement fraction
AP: Arterial phase
AUC: Area under the curve
CI: Confidence interval
IoD: Iodine density
MonoE: Monoenergetic images
nIoD: Normalized iodine density
POC: Postoperative complications
ROC: Receiver-operating characteristic
ROI: Region of interest
SBI: Spectral-based-images
SFA: Subcutaneous fat area
VFA: Visceral fat area
VP: Venous phase
Z-Eff: Effective atomic number
